# Aspiration thrombectomy with the Penumbra System for patients with stroke and late onset to treatment: a subset analysis of the COMPLETE registry

**DOI:** 10.3389/fneur.2023.1239640

**Published:** 2023-09-14

**Authors:** Ameer E. Hassan, Johanna T. Fifi, Osama O. Zaidat

**Affiliations:** ^1^Department of Neurology, University of Texas Rio Grande Valley, Valley Baptist Medical Center, Harlingen, TX, United States; ^2^Department of Neurosurgery, Icahn School of Medicine at Mount Sinai, New York, NY, United States; ^3^Department of Endovascular Neurosurgery, Mercy Health St. Vincent Medical Center, Toledo, OH, United States

**Keywords:** aspiration thrombectomy, clinical effectiveness, clinical study, functional independence, ischemic stroke, mortality, thrombectomy, time-to-treatment

## Abstract

**Background:**

The purpose of this study was to report the safety and performance of aspiration thrombectomy with the Penumbra System for patients with acute ischemic stroke (AIS) due to anterior circulation large vessel occlusion (LVO) and late onset to treatment.

**Methods:**

This is a retrospective subset analysis of a global prospective multicenter registry (COMPLETE) that enrolled adults with AIS due to LVO and a pre-stroke modified Rankin Scale score (mRS) of 0 or 1 who were treated first-line with aspiration thrombectomy either alone (A Direct Aspiration First Pass Technique [ADAPT]) or in combination with the 3D Revascularization Device (ADAPT + 3D). This subset analysis included all patients in the registry who had anterior circulation LVO, an Alberta Stroke Program Early CT Score of at least 6, and late onset to treatment (>6 h from stroke onset to puncture).

**Results:**

Of the 650 patients in the COMPLETE registry, 167 were included in this subset analysis. The rate of successful revascularization (modified thrombolysis in cerebral infarction score 2b-3 achieved) at the end of the procedure was 83.2%, the rate of good functional outcome (mRS 0–2) at 90 days was 55.4%, and the all-cause mortality rate at 90 days was 14.4%. No device-related serious adverse events (SAEs) occurred. Procedure-related SAEs occurred in 9 patients (5.4%) within 24 h and in 12 patients (7.2%) overall. The rate of successful revascularization was higher for patients treated first-line with ADAPT (88.0%) than for patients treated first-line with ADAPT + 3D (75.0%; *p* = 0.035); no significant difference was observed between the ADAPT and ADAPT + 3D groups for any other primary or secondary outcome.

**Conclusion:**

For patients with AIS due to anterior circulation LVO and with late onset to treatment, aspiration thrombectomy with the Penumbra System appears to be safe and effective. The rates of good functional outcome and all-cause mortality from this study compared favorably with those rates from the medical management arms of the DAWN and DEFUSE-3 studies.

**Clinical trial registration:**

https://www.clinicaltrials.gov, NCT03464565.

## Introduction

In patients with acute ischemic stroke (AIS), a longer time from symptom onset to treatment, and thus a longer brain ischemia time, corresponds to a worse outcome (1). Additionally, with a longer time to treatment, the thrombus becomes more organized and potentially more compacted and adherent to the vessel wall (2–4). These thrombus characteristics could increase the difficulty, number of passes, and procedure time of mechanical thrombectomy (5, 6), thus increasing the chance of a complication. On the basis of 2 randomized clinical trials [RCTs; the DAWN (7) and DEFUSE-3 (8) studies] that compared mechanical thrombectomy plus medical management versus medical management alone in patients with AIS due to anterior circulation large vessel occlusion (LVO) and with a symptom onset to treatment or randomization time of greater than 6 h, the current United States (9), European (10), and Society of Vascular and Interventional Neurology (11) guidelines recommend treatment with mechanical thrombectomy in highly selected patients (per the DAWN and DEFUSE-3 studies) who present between 6 and 24 h after symptom onset.

Two recent studies have been published on patients with AIS due to anterior circulation LVO and late onset to treatment who were treated with mechanical thrombectomy, with aspiration thrombectomy used in some but not all patients (12, 13). Neither study observed a significant difference in outcomes between patients with early versus late onset to treatment (12, 13). No previous studies are available that report the results of aspiration thrombectomy in all patients for the treatment of AIS with late onset to treatment. The purpose of this study was to report the safety and performance of aspiration thrombectomy with the Penumbra System (Penumbra, Inc., Alameda, CA) for patients with AIS due to anterior circulation LVO and with late onset to treatment, in a real-world setting.

## Methods

This study is a retrospective subset analysis of a global prospective multicenter observational registry that included patients who presented with either anterior or posterior LVO and were eligible for aspiration thrombectomy using the Penumbra System including the Penumbra 3D Revascularization Device (Penumbra, Inc.). This registry, COMPLETE (International Acute Ischemic Stroke Registry with the Penumbra System Aspiration Including the 3D Revascularization Device), was registered with ClinicalTrials.gov (NCT03464565). The study and the informed consent process were approved by the Institutional Review Board/Ethics Committee (IRB/EC) for each participating center (Supplementary Table S1). The enrollment period was July 2018 through October 2019, and 90-day follow-up was completed January 2020. This subset analysis included all patients in the registry who had anterior circulation LVO, an Alberta Stroke Program Early CT Score (ASPECTS) of at least 6, and late onset to treatment (>6 h from stroke onset to puncture).

The study protocol for the COMPLETE registry is described in detail elsewhere (14). Patients were included if they were at least 18 years old, had a pre-stroke modified Rankin Scale score (mRS) of 0 or 1, experienced AIS secondary to intracranial LVO and eligible for mechanical thrombectomy using the Penumbra System, had planned first-line treatment with the Penumbra System, and had signed informed consent per the center’s IRB/EC. Neither DAWN nor DEFUSE-3 criteria were inclusion criteria for this study. Enrolled patients were treated first-line with aspiration thrombectomy either alone (A Direct Aspiration First Pass Technique [ADAPT]) or in combination with the 3D Revascularization Device (ADAPT + 3D) as decided by the treating physician. Primary outcomes were good functional outcome (mRS 0–2) at 90 days, all-cause mortality at 90 days, and successful revascularization (modified thrombolysis in cerebral infarction score [mTICI] 2b–3 achieved) at the end of the procedure. Secondary outcomes were successful revascularization after the first pass, device-related and procedure-related serious adverse events (SAEs), embolization in new or uninvolved territory as seen on the final angiogram, symptomatic intracranial hemorrhage (sICH) within 24 h (defined as 24-h evidence of an intracranial hemorrhage [defined by using the European Cooperative Acute Stroke Study classification] associated with a worsening of the National Institutes of Health Stroke Scale score [NIHSS] of at least 4 points from baseline), vessel perforation, and vessel dissection. Imaging findings were evaluated by a core lab, and clinical events related to safety endpoints were adjudicated by independent medical reviewers.

Data analyses were performed by using SAS (version 9.4, SAS Institute, Cary, NC). Descriptive statistics were calculated for all patients and for the subgroups of patients treated first-line with ADAPT and patients treated first-line with ADAPT + 3D. The ADAPT and ADAPT + 3D groups were also compared by using the 2-tailed Mann–Whitney test or Fisher exact test as appropriate to calculate *p* values.

## Results

Of the 650 patients in the COMPLETE registry, 525 had an anterior LVO and an ASPECTS of at least 6 [Cohort A in the full COMPLETE registry results (14)]; of those 525 patients, 167 had a late onset to treatment time (>6 h) and were thus included in this subset analysis (Table 1). Median patient age for the whole late window subset was 70  years (IQR 61–78 y), and 94 patients (56.3%) were female. The first-line procedural technique was ADAPT in 100 patients, ADAPT + 3D in 64 patients, and other (Sofia Catheter, MicroVention, Inc., Aliso Viejo, CA) in 3 patients. No significant difference was observed between the ADAPT and ADAPT + 3D groups for any baseline characteristics except for the time from admission to arterial puncture, which was longer for the ADAPT + 3D group (99.0 min [IQR 52.5–146.5 min]) than for the ADAPT group (66.0 min [IQR 43.0–103.0 min]; *p* = 0.011).

**Table 1:** Baseline characteristics for patients treated with aspiration thrombectomy with the Penumbra System for AIS due to anterior circulation LVO and with late onset to treatment (>6 h from stroke onset to puncture).

	First-line procedural technique			*P* value^*^
	ADAPT ± 3D (*N* = 164)	ADAPT (*N* = 100)	ADAPT ± 3D (*N* = 64)	
Demographics				
Age, years	70.0 [61.0–78.0]	73.0 [63.0–78.5]	67.0 [58.0–78.5]	0.26
Female	56.3%	62.0%	48.4%	0.11
Race (collected only for patients in the United States)				
Asian	1.7% (2/119)	3.3% (2/60)	0.0% (0/56)	0.13
Black or African American	15.1% (18/119)	10.0% (6/60)	21.4% (12/56)	
Other (Dominican)	0.8% (1/119)	1.7% (1/60)	0.0% (0/56)	
White	79.0% (94/119)	83.3% (50/60)	73.2% (41/56)	
Not reported	3.4% (4/119)	1.7% (1/60)	5.4% (3/56)	
Ethnicity (collected only for patients in the United States)				
Hispanic or Latino	9.2% (11/119)	5.0% (3/60)	14.3% (8/56)	0.09
Not Hispanic or Latino	88.2% (105/119)	90.0% (54/60)	85.7% (48/56)	
Not reported or unknown	2.5% (3/119)	5.0% (3/60)	0.0% (0/56)	0.24
Medical history				
Previous ischemic stroke	16.2%	17.0%	15.6%	>0.99
Previous transient ischemic attack	8.4%	9.0%	6.3%	0.77
Previous hemorrhagic stroke	0.6%	1.0%	0.0%	>0.99
Previous intracranial surgery	1.2%	1.0%	1.6%	>0.99
Cardiovascular/vascular disease	47.9%	52.0%	42.2%	0.26
Atrial fibrillation	28.7%	34.0%	21.9%	0.19
Diabetes	25.1%	23.0%	28.1%	0.47
Renal failure	5.4%	6.0%	4.7%	>0.99
Hypertension	71.9%	67.0%	81.3%	0.0503
Hyperlipidemia	43.7%	41.0%	48.4%	0.42
Seizures	0.6%	0.0%	1.6%	0.39
Atherosclerosis	12.6%	14.0%	9.4%	0.47
Headaches/migraines	2.4%	2.0%	3.1%	0.64
Hematologic disorder	6.0%	5.0%	7.8%	0.51
Tobacco use (current or former)	32.9%	33.0%	32.8%	>0.99
Stroke onset determination				
Witnessed	22.9% (38/166)	22.2% (22/99)	25.0%	0.17
Present upon wakeup	25.9% (43/166)	30.3% (30/99)	17.2%	
Unwitnessed; time last seen well	51.2% (85/166)	47.5% (47/99)	57.8%	
Transferred from another hospital	64.7%	62.0%	70.3%	0.23
Time from stroke onset to hospital admission, h	8.6 [6.1–13.1]	8.6 [6.1–13.2]	8.7 [6.1–12.7]	0.95
Intravenous tPA given before procedure	21.6%	23.0%	20.3%	0.85
Transferred from another hospital	83.3% (30/36)	78.3% (18/23)	92.3% (12/13)	0.39
Direct admission	16.7% (6/36)	21.7% (5/23)	7.7% (1/13)	
Pre-treatment imaging modality^†^				
Noncontrast CT	91.0%	91.0%	90.6%	>0.99
CT perfusion	57.5%	57.0%	57.8%	>0.99
MRI	4.8%	7.0%	1.6%	0.15
Target vessel location^†^				
Internal carotid artery	5.4%	5.0%	6.3%	0.74
Carotid T	11.4%	10.0%	12.5%	0.62
M1 MCA	57.5%	56.0%	59.4%	0.75
M2 MCA	23.4%	25.0%	21.9%	0.71
Other anterior^‡^	2.4%	4.0%	0.0%	0.16
Left-sided occlusion^§^	53.9%	54.0%	53.1%	>0.99
Pre-stroke modified Rankin Scale score				
0	70.7%	69.0%	71.9%	0.80
1	28.1%	30.0%	26.6%	
2	1.2%	1.0%	1.6%	
National Institutes of Health Stroke Scale score	12.0 [7.0–17.0]	11.0 [7.5–17.0]	12.0 [7.0–17.5]	0.50
Alberta Stroke Program Early CT Score^§^	8.0 [7.0–9.0]	8.0 [7.0–9.0]	8.0 [7.0–9.0]	0.69
Time from stroke onset to arterial puncture, h	10.5 [7.6–14.8]	10.4 [7.4–14.4]	10.6 [7.9–15.3]	0.48
Time from admission to arterial puncture,^||^ min	71.5 [47.0–120.5] (*n* = 164)	66.0 [43.0–103.0] (*n* = 97)	99.0 [52.5–146.5]	0.011

Continuous variables are reported as median [IQR] and categorical variables are reported as percentage. The ADAPT and ADAPT + 3D groups were also compared by using the 2-tailed Mann–Whitney test or Fisher exact test as appropriate to calculate *p* values. Another first-line procedural technique was used for 3 patients; those patients were excluded from comparison. 3D, 3D Revascularization Device; ADAPT, A Direct Aspiration First Pass Technique; AIS, acute ischemic stroke; CT, computerized tomography; LVO, large vessel occlusion; MCA, middle cerebral artery; MRI, magnetic resonance imaging; tPA, tissue plasminogen activator. ^*^Please use caution in interpreting *p* values as this analysis was not powered for the subgroups. ^†^More than one response allowed. ^‡^M3 MCA, M4 MCA, or anterior cerebral artery. ^§^Core lab–evaluated otherwise treating physician–evaluated. ^||^Patients with pre-stroke admission were excluded from this calculation.

No significant difference was observed between the ADAPT and ADAPT + 3D groups for the number of passes performed or for the median time from stroke onset to mTICI 2b–3 or final angiogram (Table 2). The median time from arterial puncture to mTICI 2b–3 or final angiogram for the whole late window subset was 28.0 min; the time was longer for the ADAPT + 3D group (39.0 min [IQR 20.5–61.5 min]) than for the ADAPT group (24.0 min [IQR 15.5–40.5 min]; *p* = 0.002).

**Table 2:** Procedural information for patients treated with aspiration thrombectomy with the Penumbra System for AIS due to anterior circulation LVO and with late onset to treatment (>6 h from stroke onset to puncture).

	First-line procedural technique			*P* value^*^
	ADAPT ± 3D (*N* = 164)	ADAPT (*N* = 100)	ADAPT ± 3D (*N* = 64)	
First-line procedural technique				
ADAPT	59.9%	100%	0%	NA
ADAPT + 3D	38.3%	0%	100%	
Other^†^	1.8%	0%	0%	
Number of passes				
1	38.3%	41.0%	34.4%	0.16
2	21.6%	24.0%	17.2%	
3	20.4%	21.0%	20.3%	
4 or more	19.8%	14.0%	28.1%	
Time from stroke onset to mTICI 2b–3 or final angiogram, h^‡^	11.1 [8.4–15.7]	10.8 [8.0–15.1]	11.1 [8.5–15.9]	0.36
Time from arterial puncture to mTICI 2b–3 or final angiogram, min^‡^	28.0 [17.0–47.0]	24.0 [15.5–40.5]	39.0 [20.5–61.5]	0.002

Continuous variables are reported as median [IQR] and categorical variables are reported as percentage. The ADAPT and ADAPT + 3D groups were also compared by using the 2-tailed Mann–Whitney test or Fisher exact test as appropriate to calculate *p* values. Another first-line procedural technique was used for 3 patients; those patients were excluded from comparison. 3D, 3D Revascularization Device; ADAPT, A Direct Aspiration First Pass Technique; AIS, acute ischemic stroke; LVO, large vessel occlusion; mTICI, modified treatment in cerebral infarction score; NA, not applicable. ^*^Please use caution in interpreting *p* values as this analysis was not powered for the subgroups. ^†^Sofia Catheter. ^‡^Core lab–evaluated.

The rate of successful revascularization at the end of the procedure for the whole late window subset was 83.2%; the rate was significantly different between the ADAPT (88.0%) and ADAPT + 3D (75.0%) groups (*p* = 0.035; Table 3). The rate of good functional outcome at 90 days for the whole late window subset was 55.4%; no significant difference was observed between the rates of the ADAPT (58.3%) and ADAPT + 3D (50.0%) groups (*p* = 0.32). The all-cause mortality rate at 90 days for the whole late window subset was 14.4%; no significant difference was observed between the rates of the ADAPT (13.0%) and ADAPT + 3D (17.2%) groups (*p* = 0.50).

**Table 3:** Outcomes for patients treated with aspiration thrombectomy with the Penumbra System for AIS due to anterior circulation LVO and with late onset to treatment (>6 h from stroke onset to puncture).

	First-line procedural technique			*P* value^*^
	ADAPT ± 3D (*N* = 164)	ADAPT (*N* = 100)	ADAPT ± 3D (*N* = 64)	
Primary outcomes				
mTICI 2b–3 at the end of the procedure^†^	83.2% [76.7–88.6%]	88.0% [80.0–93.6%]	75.0% [62.6–85.0%]	0.035
mRS 0–2 at 90 days	55.4% (87/157) [47.3–63.3%]	58.3% (56/96) [47.8–68.3%]	50.0% (29/58) [36.6–63.4%]	0.32
All-cause mortality at 90 days	14.4% [9.4–20.6%]	13.0% [7.1–21.2%]	17.2% [8.9–28.7%]	0.50
Secondary outcomes, by patient				
mTICI 2b–3 after the first pass^†^	49.1% (81/165) [41.2–57.0%]	51.0% [40.8–61.1%]	46.8% (29/62) [34.0–59.9%]	0.63
Device-related SAEs within 24 h^‡^	0.0% [0.0–2.2%]	0.0% [0.0–3.6%]	0.0% [0.0–5.6%]	NA
Device-related SAEs, all^‡^	0.0% [0.0–2.2%]	0.0% [0.0–3.6%]	0.0% [0.0–5.6%]	NA
Procedure-related SAEs within 24 h	5.4% [2.5–10.0%]	7.0% [2.9–13.9%]	3.1% [0.4–10.8%]	0.48
Procedure-related SAEs, all^‡^	7.2%^§^ [3.8–12.2%]	9.0% [4.2–16.4%]	4.7% [1.0–13.1%]	0.37
Embolization in new or uninvolved territory at end of procedure^†^	2.4% [0.7–6.0%]	1.0% [0.0–5.4%]	4.7% [1.0–13.1%]	0.30
Symptomatic intracranial hemorrhage within 24 h^‡^	4.2% [1.7–8.4%]	5.0% [1.6–11.3%]	3.1% [0.4–10.8%]	0.71
Vessel perforation^||^	0.0% [0.0–2.2%]	0.0% [0.0–3.6%]	0.0% [0.0–5.6%]	NA
Vessel dissection^||^	0.6% [0.0–3.3%]	1.0% [0.0–5.4%]	0.0% [0.0–5.6%]	>0.99

Variables are reported as percentage [95% CI]. The ADAPT and ADAPT + 3D groups were compared by using the 2-tailed Fisher exact test to calculate *p* values. Another first-line procedural technique was used for 3 patients; those patients were excluded from comparison. 3D, 3D Revascularization Device; ADAPT, A Direct Aspiration First Pass Technique; AIS, acute ischemic stroke; LVO, large vessel occlusion; mRS, modified Rankin Scale score; mTICI, modified treatment in cerebral infarction score; NA, not applicable; SAE, serious adverse event. ^*^Please use caution in interpreting *p* values as this analysis was not powered for the subgroups. ^†^Core lab–evaluated otherwise treating physician–evaluate. ^‡^Independent medical reviewer–adjudicated. ^§^13 events: 6 events of intracranial hemorrhage and 1 event each of anemia, subarachnoid hemorrhage, vascular access site hematoma, cerebral infarction, stroke in evolution, pulmonary edema, and respiratory arrest. ^||^Independent medical reviewer–adjudicated otherwise treating physician–evaluated.

For the whole late window subset, the rate of successful revascularization after the first pass was 49.1% (Table 3). No device-related SAEs occurred. Procedure-related SAEs occurred in 9 patients (5.4%) within 24 h and in 12 patients (7.2%) overall. Embolization in new or uninvolved territory at the end of the procedure occurred in 4 patients (2.4%) and sICH within 24 h occurred in 7 patients (4.2%). Vessel perforation occurred in 0 patients and vessel dissection occurred in 1 patient (0.6%). No significant difference was observed between the ADAPT and ADAPT + 3D groups for the rate of any secondary outcome.

## Discussion

Patients with AIS due to anterior circulation LVO and with late onset to treatment can be effectively treated with aspiration thrombectomy. Late onset to treatment patients selected per the DAWN and DEFUSE-3 advanced imaging criteria are more likely to benefit from mechanical thrombectomy (15), and current guidelines on treating late onset to treatment stroke patients with mechanical thrombectomy (9–11) are based on the results of the DAWN and DEFUSE-3 studies. However, other stroke patients with late onset to treatment can benefit from mechanical thrombectomy, including those patients with signs of less severe stroke, especially with a higher ASPECTS (12, 13, 16, 17) or good collateral status (16, 18, 19). In this study, patients with late onset to treatment, anterior circulation LVO, and an ASPECTS of at least 6 had a high rate of successful revascularization, a high rate of good functional outcome at 90 days, and a low mortality rate at 90 days. Furthermore, most outcomes were similar between the patients treated with ADAPT and the patients treated with ADAPT + 3D. This study presents real-world experience of treating patients with AIS due to anterior circulation LVO and with late onset to treatment.

The rates of good functional outcome at 90 days and of all-cause mortality at 90 days from this study compared favorably with those rates from the medical management arms of the DAWN (7) and DEFUSE-3 (8) studies (Table 4). The rate of good functional outcome at 90 days was considerably higher in the current study (55.4%) than in the medical management arms of the DAWN (13.1%) and DEFUSE-3 (16.7%) studies, and the all-cause mortality rate at 90 days was lower in the current study (14.4%) than in the medical management arms of the DAWN (18.2%) and DEFUSE-3 (25.6%) studies. Additionally, the rate of good functional outcome at 90 days was higher in the current study (55.4%) than in the stent retriever thrombectomy arm of the DAWN study (48.6%) and the mechanical thrombectomy arm of the DEFUSE-3 study (44.6%). The all-cause mortality rate at 90 days in the current study (14.4%) was comparable to the rates in the stent retriever thrombectomy arm of the DAWN study (18.7%) and the mechanical thrombectomy arm of the DEFUSE-3 study (14.1%), and the sICH rate in the current study (4.2%) was comparable to the rates in the stent retriever thrombectomy arm of the DAWN study (5.6%) and the mechanical thrombectomy arm of the DEFUSE-3 study (6.5%).

**Table 4:** Comparison of the current study (from the COMPLETE registry) with the DAWN (7) and DEFUSE-3 (8) studies, for patients with AIS due to anterior circulation LVO and with late onset to treatment who were treated with mechanical thrombectomy plus medical management or with medical management alone.

Study or registry name	Treatment	No. patients	Outcomes		
			mRS 0–2 at 90 days	All-cause mortality at 90 days	sICH
COMPLETE	ADAPT ± 3D	167	55.4% (87/157)	14.4%	4.2%
	ADAPT	100	58.3% (56/96)	13.0%	5.0%
	ADAPT + 3D	64	50.0% (29/58)	17.2%	3.1%
DAWN (7)	Stent retriever thrombectomy + medical management	107	48.6%	18.7%	5.6%
	Medical management	99	13.1%	18.2%	3.0%
	*P* value	---	>0.99^*^	NSD	NSD
DEFUSE-3 (8)	Mechanical thrombectomy + medical management	92	44.6%	14.1%	6.5%
	Medical management	90	16.7%	25.6%	4.4%
	*P* value	---	<0.001	0.05	0.75

3D, 3D Revascularization Device; ADAPT, A Direct Aspiration First Pass Technique; AIS, acute ischemic stroke; LVO, large vessel occlusion; mRS, modified Rankin Scale score; NSD, no significant difference; sICH, symptomatic intracranial hemorrhage. ^*^Posterior probability of superiority.

In the present study, no significant difference was observed between the ADAPT and ADAPT + 3D groups for the rates of good functional outcome at 90 days, all-cause mortality at 90  days, sICH within 24 h, and device-related SAEs within 24 h (Table 3). Likewise, an RCT of ADAPT versus ADAPT + 3D (Penumbra Separator 3D trial [3-D trial]), in which patients were included if they presented within 8h, also observed no significant differences between the groups for those variables (20). In contrast to the present study, that RCT reported that the rate of successful revascularization at the end of the procedure was higher for ADAPT + 3D (81.9%) than for ADAPT (69.8%; 90% CI 2.0–22.2%).

The outcomes from the current study were in range of the outcomes reported by other studies of late onset to treatment in patients with AIS due to anterior circulation LVO (Supplementary Table S2) (7, 8, 12, 13, 16, 17, 21–26). The rate of good functional outcome was higher for most mechanical thrombectomy study arms or studies (including the current study) than for the all the medical management arms among the studies. Heterogeneity among these studies in regard to study design, baseline NIHSS, imaging and subsequent patient selection, percentage of patients with M2 middle cerebral artery occlusion, intravenous tissue plasminogen activator given before the procedure, and treatment modality limits direct comparisons between them. In particular, the median NIHSS was appreciably lower and the rate of intravenous tissue plasminogen activator given before the procedure was appreciably higher in the current study than in the DAWN (7) and DEFUSE-3 (8) studies.

A limitation of this study is that it was a *post hoc* analysis of prospectively collected data. This limitation is reflective of the real-world situation in which the data for this study were collected. The statistical analysis was not powered for the ADAPT and ADAPT + 3D groups and patients were not randomly assigned between those groups. However, most baseline characteristics were similar between the 2 groups (Table 1). The exception was that the median time from admission to arterial puncture was longer for the ADAPT + 3D group (99 min) than for the ADAPT group (66 min); however, no significant difference was observed between the 2 groups for the time from stroke onset to arterial puncture. The median time from arterial puncture to mTICI 2b–3 or final angiogram was longer for the ADAPT + 3D group (39 min) than for the ADAPT group (24 min); however, the 3-D trial, in which patients were included if they presented within 8 h, also reported that the median time from arterial puncture to mTICI 2b–3 was longer for the ADAPT + 3D group (49 min) than for the ADAPT group (39 min) (20). In the present study, the rate of successful revascularization at the end of procedure was higher for the ADAPT group than for the ADAPT + 3D group, whereas in the 3-D trial, the opposite was observed (20). Changes over time in the thrombus composition and its adherence to the vessel wall may influence the optimal AIS treatment choice during a later time window. Participating centers might have measured infarcted brain tissue by using advanced imaging (per DAWN or DEFUSE-3 guidelines) to determine patient eligibility as part of standard of care; however, neither DAWN nor DEFUSE-3 criteria were inclusion criteria for this study. The study population was limited to patients with anterior circulation LVO and an ASPECTS of at least 6; however, other studies on AIS with late onset to treatment have similarly limited their study population to patients with anterior circulation LVO, and most of those studies [excepting 1 study on patients with an ASPECTS of 5 or less (25) and the DAWN study, which did not report ASPECTS (7)] reported a median ASPECTS that was identical to or at most 1 unit higher than that reported in the current study (Supplementary Table S2) (8, 12, 13, 16, 17, 21–24, 26).

In conclusion, for patients with AIS due to anterior circulation LVO and with late onset to treatment, aspiration thrombectomy with the Penumbra System was safe and effective, and results were similar for patients treated with ADAPT and patients treated with ADAPT + 3D.

## Data availability statement

The raw data supporting the conclusions of this article will be made available by the authors, without undue reservation.

## Ethics statement

The studies involving humans were approved by the IRBs/ECs listed in Supplementary Table S1. The studies were conducted in accordance with the local legislation and institutional requirements. The participants provided their written informed consent to participate in this study.

## Author contributions

All authors listed have made a substantial, direct, and intellectual contribution to the work and approved it for publication.

## Funding

This study was funded by Penumbra, Inc.

## Conflict of interest

AH reports consultant/speaker fees from Medtronic, Microvention, Stryker, Penumbra, Cerenovus, Genentech, GE Healthcare, Scientia, Balt, *Viz.*ai, Insera Therapeutics, Proximie, NeuroVasc, NovaSignal, Vesalio, Rapid Medical, Imperative Care, and Galaxy Therapeutics; principal investigator for COMPLETE study (Penumbra), LVO SYNCHRONISE (*Viz.*ai), Millipede Stroke Trial (Perfuze), and RESCUE – ICAD (Medtronic); steering committee/publication committee member for SELECT, DAWN, SELECT 2, EXPEDITE II, EMBOLISE, CLEAR, ENVI, DELPHI, and DISTALS; and DSMB for the COMAND trial. JF reports personal fees from Penumbra, Stryker, Microvention, and Cerenovus; ownership interest in Imperative Care; and grants from *Viz.*ai. OZ reports grants and personal fees from Penumbra and four other companies and patents pending or issued for aneurysm and stroke device(s). The authors declare that this study received funding from Penumbra, Inc. The funder had the following involvement in the study: AH, JF, and OZ were the study’s principal investigators and were involved in study design. The funder was responsible for database setup, site monitoring, and data management and provided statistical, writing, and editorial support.

## Publisher’s note

All claims expressed in this article are solely those of the authors and do not necessarily represent those of their affiliated organizations, or those of the publisher, the editors and the reviewers. Any product that may be evaluated in this article, or claim that may be made by its manufacturer, is not guaranteed or endorsed by the publisher.

## Abbreviations

3D: 3D Revascularization Device
ADAPT: A Direct Aspiration First Pass Technique
AIS: Acute ischemic stroke
ASPECTS: Alberta Stroke Program Early CT Score
IRB/EC: Institutional Review Board/Ethics Committee
LVO: Large vessel occlusion
MCA: Middle cerebral artery
mRS: modified Rankin Scale score
mTICI: modified thrombolysis in cerebral infarction score
NIHSS: National Institutes of Health Stroke Scale score
sICH: Symptomatic intracranial hemorrhage
