# Supplementary material for: Aspiration thrombectomy with the Penumbra System for patients with stroke and late onset to treatment: a subset analysis of the COMPLETE registry
Source: Front Neurol. 2023 Sep 14;14:1239640. doi: 10.3389/fneur.2023.1239640 (PMC10546392; doi:10.3389/fneur.2023.1239640)
Supplement: Supplementary file 1 [file Table_1.pdf]

Supplemental Table S1. COMPLETE Study IRB/EC names and numbers.

Center	IRB/EC Name	IRB/EC Number
Universitätsklinikum Erlangen<br>Schwabachanlage 6<br>91054<br>Erlangen<br>Germany	Ethikkommission der Friedrich-Alexander-Universität Erlangen-Nürnberg<br>Krankenhausstraße 12<br>91054<br>Erlangen<br>Germany	355_18 Bc
AdventHealth Orlando<br>601 E. Rollins Street<br>Orlando, FL 32803<br>USA	AdventHealth Orlando IRB<br>901 N. Lake Destiny Road, Suite 400<br>Maitland, FL 32751<br>USA	1313681-2
Radiology Imaging Associates, P.C.<br>501 E. Hampden Avenue<br>Englewood, CO 80113<br>USA	HCA – HealthONE IRB<br>4900 South Monaco Street, Suite 220<br>Denver, CO 80237<br>USA	1335634-9
Universitätsklinikum Schleswig-Holstein Campus Lübeck<br>Ratzeburger Allee 160<br>23538<br>Lübeck<br>Germany	Ethikkommission zu Lübeck - Universität zu Lübeck Ratzeburger Allee 160<br>Haus 2<br>23538<br>Lübeck<br>Germany	18-142
Northwell Health<br>300 Community Drive<br>Manhasset, NY 11030<br>USA	WCG IRB<br>1019 39th Ave SE, Suite 120<br>Puyallup, WA 98374<br>USA	1-1159938-1
Fort Sanders Regional Medical Center<br>1901 West Clinch Avenue<br>Knoxville, TN 37916<br>USA	Covenant Health IRB<br>280 Fort Sanders West Blvd. Bldg. 4, Suite 205<br>Knoxville, TN 37922<br>USA	2018-285
Palmetto Health Richland Hospital<br>5 Richland Medical Park Drive<br>Columbia, SC 29203<br>USA	Prisma Health-Midlands IRB<br>5 Richland Medical Park Drive<br>Columbia, SC 29203<br>USA	Pro00082519

Center	IRB/EC Name	IRB/EC Number
Houston Methodist Hospital<br>6565 Fannin Street<br>Houston, TX 77030<br>USA	Houston Methodist Research Institute IRB<br>6670 Bertner Avenue<br>Houston, TX 77030<br>USA	Pro00020259
Cedar Sinai Medical Center<br>8700 Beverly Boulevard<br>Los Angeles, CA 90048<br>USA	Cedars-Sinai, Office of Research Compliance and Quality Improvement<br>6500 Wilshire Blvd., Suite 1800<br>Los Angeles, CA 90048<br>USA	Pro00056399
University of Kansas Medical Center Research Institute<br>3901 Rainbow Boulevard<br>Kansas City, KS 66160<br>USA	KUMC (University of Kansas Medical Center)<br>Human Research Protection Program IRB<br>Mail-Stop 1032<br>3901 Rainbow Blvd.<br>Kansas City, KS 66160<br>USA	STUDY00143286
Mount Sinai Medical Center<br>1450 Madison Avenue<br>Klingenstein Clinical Center (KCC)<br>1 North<br>New York, NY 10029<br>USA	WCG IRB<br>1019 39th Ave SE, Suite 120<br>Puyallup, WA 98374<br>USA	1-1095711-1
McAllen Medical Center<br>301 West Expressway 83<br>McAllen, TX 78503<br>USA	WCG IRB<br>1019 39th Ave SE, Suite 120<br>Puyallup, WA 98374<br>USA	1-1163145-1
Yale New Haven Hospital<br>2 Church Street South, Suite 401<br>New Haven, CT 06519<br>USA	WCG IRB<br>1019 39th Ave SE, Suite 120<br>Puyallup, WA 98374<br>USA	1-1104399-1
Erlanger Medical Center<br>975 East 3rd Street<br>Box 376<br>Chattanooga, TN 37403<br>USA	UT (University of Tennessee)-College of Medicine IRB<br>960 East Third St, Suite 100<br>Chattanooga, TN 37403<br>USA	Not Available

Center	IRB/EC Name	IRB/EC Number
Jackson Memorial Hospital<br>1611 NW 12th Avenue<br>Miami, FL 33136<br>USA	University of Miami<br>Human Subject Research Office (M809)<br>1400 NW 10th Avenue, Suite 1200A<br>Miami, FL 33136<br>USA	20181175
The Valley Hospital<br>223 N. Van Dien Avenue<br>Ridgewood, NJ 07450<br>USA	WCG IRB<br>1019 39th Ave SE, Suite 120<br>Puyallup, WA 98374<br>USA	1-1096004-1
Brigham & Women's Hospital<br>75 Francis Street<br>Boston, MA 02115<br>USA	Human Research Committee<br>Partners HealthCare Systems, Inc.<br>399 Revolution Drive, Suite 710<br>Somerville, MA 02145<br>USA	2018P001818
Foundation Ophthalmic Adolphe De Rothschild<br>29 Rue Manin<br>75019<br>Paris<br>France	C.P. P Sud-Ouest et Outre-Mer IV<br>Cabanis Haut-Centre Hospitalier ESQUIROL<br>15 rue du Docteur MARCLAND<br>872025<br>LIMOGES<br>France	CPP2019-01'-008/2018-A03346-49
Swedish Medical Center-Cherry Hill<br>550-17th Avenue<br>Seattle, WA 98122<br>USA	WCG IRB<br>1019 39th Ave SE, Suite 120<br>Puyallup, WA 98374<br>USA	1-1169659-1
Thomas Jefferson University Hospital<br>125 S. 9th Street<br>Sheridan Building<br>Philadelphia, PA 19107<br>USA	WCG IRB<br>1019 39th Ave SE, Suite 120<br>Puyallup, WA 98375<br>USA	1-1123999-1
Los Robles Hospital<br>215 W Janss Road<br>Thousand Oaks, CA 91360<br>USA	WCG IRB<br>1019 39th Ave SE, Suite 120<br>Puyallup, WA 98376<br>USA	1-1091773-1

Center	IRB/EC Name	IRB/EC Number
Semmes Murphey<br>Methodist University Hospital<br>1265 Union Avenue<br>Memphis, TN 38104<br>USA	UTHSC (University of Tennessee Health Science Center) IRB<br>910 Madison Avenue, Suite 600<br>Memphis, TN 38163<br>USA	18-06397-XP
Mercy San Juan Medical Center<br>6501 Coyle Avenue<br>Carmichael, CA 95608<br>USA	Dignity Health IRB<br>3400 Data Drive<br>Rancho Cordova, CA 95670<br>USA	00006573
SSM St. Clare Healthcare<br>1015 Bowles Avenue<br>Fenton, MO 63026<br>USA	SSMSTL (SSM Health Care St Louis) IRB<br>1015 Corporate Square Dr. #150<br>St Louis, MO 63132<br>USA	18-06-1278
St Jude Medical Center<br>101 E. Valencia Mesa Drive<br>Fullerton, CA 92835<br>USA	St Joseph Health IRB<br>3345 Michelson Dr, Suite 100<br>Irvine, CA 92612<br>USA	18-114
Ochsner Medical Center<br>1514 Jefferson Highway<br>New Orleans, LA 70121<br>USA	Ochsner Clinic Foundation IRB<br>1514 Jefferson Highway<br>New Orleans, LA 70121<br>USA	2018.252
NYU Langone Hospital - Brooklyn<br>550 First Avenue<br>New York, NY 10016<br>USA	NYU School of Medicine's Office of Science and Research Institutional Review Board 1<br>1 Park Avenue, 6th Floor<br>New York, NY 10016<br>USA	i18-01059
Eden Medical Center<br>20103 Lake Chabot Road<br>Castro Valley, CA 94546<br>USA	Sutter Health IRB<br>2121 N. California Blvd., Suite 310<br>Walnut Creek, CA 94596<br>USA	1303009-1
Valley Baptist Health System-Harlingen<br>2101 Pease Street<br>Harlingen, TX 78550<br>USA	MetroWest Medical Center IRB<br>115 Lincoln Street<br>Framingham, MA 01702<br>USA	2018-184

Center	IRB/EC Name	IRB/EC Number
Mercy Health St. Vincent Medical Center LLC.<br>2213 Cherry Street<br>Toledo, OH 43608<br>USA	Mercy Health North LLC Adult IRB Research Oversight and Education<br>2200 Jefferson Ave.<br>Toledo, OH 43604<br>USA	2018-24-MHSVM
Banner Desert Medical Center<br>1400 South Dobson Avenue<br>Mesa, AZ 85202<br>USA	WCG IRB<br>1019 39th Ave SE, Suite 120<br>Puyallup, WA 98374<br>USA	1-1161375-1
Naples Community Hospital<br>50 7th Street N.<br>Naples, FL 34102<br>USA	NCH_IRB<br>350 Seventh Street N.<br>Naples, FL 34102<br>USA	Not Available
Universitätsklinikum Magdeburg A. ö. R.<br>Leipziger Str. 44<br>39120<br>Magdeburg<br>Germany	Ethik-Kommission der Otto-von-Guericke-Universität an der Medizinischen Fakultät und am Universitätsklinikum Magdeburg A.ö.R<br>Leipziger Str. 44<br>39120<br>Magdeburg<br>Germany	178/18
CHU de Bordeaux<br>Hôpital Pellegrin<br>Place Amélie Raba Léon<br>33000<br>Bordeaux<br>France	C.P. P Sud-Ouest et Outre-Mer IV<br>Cabanis Haut<br>Centre Hospitalier ESQUIROL<br>15 rue du Docteur MARCLAND<br>872025<br>LIMOGES Cedex<br>France	CPP2019-01'-008/2018-A03346-49
Charité<br>Universitätsmedizin Berlin<br>Charitéplatz 1<br>10117<br>Berlin<br>Germany	Ethikkommission der Charité<br>Universitätsmedizin Berlin<br>Campus Charité Mitte, Charitéplatz 1<br>Geländeadresse: Virchowweg 10<br>10117<br>Berlin<br>Germany	N/A

Center	IRB/EC Name	IRB/EC Number
Klinikum Chemnitz gGmbH<br>Flemmingstr	Sächsische Landesärztekammer – Ethikkommission<br>Schützenhöhe 16<br>01099<br>Dresden<br>Germany	EK-BR-69/18-1
Samodzielny Publiczny Szpital<br>Kliniczny nr 4 w Lublinie<br>ul. Jaczewskiego 8<br>20-954<br>Lublin<br>Poland	Komisja Bioetyczna przy Uniwersytecie Medycznym w Lublinie<br>Al. Racławickie 1<br>pokój 128<br>20-059<br>Lublin<br>Poland	KE-0254/86/2019
Hospital Universitari Vall d'Hebron<br>Passeig de la Vall d'Hebron 119-129<br>08035<br>Barcelona<br>Spain	Comité Ético de Investigación Clínica (CEIC) del Hospital Universitario Vall d'Hebron<br>Passeig de la Vall d'Hebron 119-129<br>Edificio Hospital Maternoinfantil, planta 13<br>08035<br>Barcelona<br>Spain	PR(AG)250/2018
Davidovsky Moscow City Hospital №23<br>Yauzskaya Ulitsa, 11<br>109240<br>Moscow<br>Russia	Independent Ethics Committee at SBHI (State Budgetary Healthcare Institution<br>CCH (City Clinical Hospital) n.a. I. V. Davydovsky MHD (Moscow Health Department)<br>11 Yauzskaya str.<br>109240<br>Moscow<br>Russia	Not Available
City Clinical Hospital No. 1 named after N. I. Pirogov<br>Leninsky Ave, 8<br>119049<br>Moscow<br>Russia	Local Ethics Committee of City Clinical Hospital No. 1 named after N. I. Pirogov<br>Leninsky Ave, 8<br>119049<br>Moscow<br>Russia	Not Available

Center	IRB/EC Name	IRB/EC Number
Multidisciplinary City Hospital №2<br>Uchebnyy Pereulok, 5<br>194354<br>St. Petersburg<br>Russia	Local Ethics Committee of SPb GBUZ City Multidisciplinary Hospital №2<br>Uchebnyy Pereulok, 5<br>194354<br>St. Petersburg<br>Russia	Not Available
Hospital Clínico Universitario Virgen de la Arrixaca<br>Ctra. Madrid-Cartagena, s/n<br>30120 Murcia (El Palmar)<br>Spain	CEIC del Hospital Virgen de la Arrixaca<br>Ctra. Madrid-Cartagena<br>Unidad AECC 1ª Planta<br>30120 El Palmar (Murcia)<br>Spain	2018-10-05-HCUVA
