# Supplementary material for: Aspiration thrombectomy with the Penumbra System for patients with stroke and late onset to treatment: a subset analysis of the COMPLETE registry
Source: Front Neurol. 2023 Sep 14;14:1239640. doi: 10.3389/fneur.2023.1239640 (PMC10546392; doi:10.3389/fneur.2023.1239640)
Supplement: Supplementary file 2 [file Table_2.pdf]

Supplemental Table S2. Comparison of patients with AIS due to anterior circulation LVO and with late onset to treatment who were treated with mechanical thrombectomy plus medical management or with medical management. Continuous variables are reported as mean (SD) or as median [IQR] and categorical variables are reported as percentage.

Citation	Study or registry name	Study design	Treatment	No. patients	Baseline characteristics							Endpoints		
					Age, years	Female	NIHSS	ASPECTS	M2 MCA occlusion	Intravenous tPA given before procedure	Time from stroke onset to arterial puncture (unless otherwise noted), h	mRS 0-2 at 90 days	All-cause mortality at 90 days	sICH
Current study	COMPLETE	Retrospective analysis of prospectively collected data	ADAPT ± 3D	167	70 [61-78]	56.3%	12 [7-17]	8 [7-9]	23.4%	21.6%	10.5 [7.6-14.8]	55.4% (87/157)	14.4%	4.2%
			ADAPT	100	73 [63-78.5]	62.0%	11 [7.5-17]	8 [7-9]	25.0%	23.0%	10.4 [7.4-14.4]	58.3% (56/96)	13.0%	5.0%
			ADAPT + 3D	64	67 [58-78.5]	48.4%	12 [7-17.5]	8 [7-9]	21.9%	20.3%	10.6 [7.9-15.3]	50.0% (29/58)	17.2%	3.1%
			P value	---	0.26	0.11	0.50	0.69	0.71	0.85	0.48	0.32	0.50	0.71
Albers et al, 2018 (8)	DEFUSE-3	RCT	Mechanical thrombectomy + medical management	92	70 [59-79]	50.0%	16 [10-20]	8 [7-9]	0.0%	10.9%	10.9 [8.8-12.4][a,b]	44.6%	14.1%	6.5%
			Medical management	90	71 [59-80]	51.1%	16 [12-21]	8 [7-9]	1.1%	8.9%	10.7 [8.7-13.1][a]	16.7%	25.6%	4.4%
			P value	---	NR	NR	NR	NR	NR	NR	NR	<0.001	0.05	0.75
Broocks et al, 2022 (25)	GSR-ET	Retrospective analysis of prospectively collected data	Endovascular thrombectomy	285	73 [62-81]	48.8% (139/285)	17 [13-19]	5 [4-5]	NR	33.3%	11.3 [8.1-14.4][c]	20.0%	35.1%	6.3%
Desai et al, 2022 (23)	-----	Retrospective analysis of prospectively collected data	Endovascular thrombectomy	86	71 (17)	57.0%	14 [11-19]	9 [8-10]	0.0%	8.1%	13.3 [8.6-19.7]	39.5%	17.4%	5.8%
Evans et al, 2018 (16)	ESCAPE	Post hoc analysis from RCT	Thrombectomy + medical management	33	66.1 [15.2]	60.6%	14 [4]	9 [2]	See footnote[d]	24.2%	7.8 [3.0][a]	48.5%	15.2%	0.0%
			Medical management	26	67.9 [21.9]	42.3%	17 [12]	8.5 [3]	See footnote[e]	57.8%	6.8 [1.8][a]	29.2% (7/24)	12.5% (3/24)	0.0%
			P value	---	NR	NR	NR	NR	NR	NR	NR	0.178	NR	>0.99
Hendrix et al., 2021 (12)	-----	Retrospective	Mechanical thrombectomy (primary aspiration, 27.3%)	132	75 [60-83]	53.8%	17 [11-22]	9 [7-10]	15.2%	13.6%	11.4 [7.9-15.3][f]	32.6%	18.9%	9.1%
Jovin et al, 2021 (21)	AURORA	Individual patient data meta-analysis	Mechanical thrombectomy + medical management	266	68.4 (13.8)	54.9%	16 [13-20]	8 [7-9][g]	2.6%	10.5%	10.5 [7.8-13.0][a] 11.0 [8.5-13.5]	45.9%	16.5%	5.3%
			Medical management	239	68.7 (13.7)	47.3%	16 [13-21]	8 [7-9][g]	3.3%	15.9%	10.3 [7.9-13.8][a]	19.3% (46/238)	19.3% (46/238)	3.3% (8/239)
			P value	---	NR	NR	NR	NR	NR	NR	NR	<0.001[h]	0.88[h]	0.23[h]
Leischner et al, 2021 (24)	GSR-ET	Retrospective analysis of prospectively collected data	Endovascular thrombectomy	30[i]	67 [63-81]	63%	15 [13-18]	9 [7-10]	0.0%	NR[j]	>6	25%[k]	25%[k]	NR
				57[l]	77 [65-83]	35%	17 [15-20]	9 [8-10]	30%	NR[j]	>6	22%[k]	26%[k]	NR
Nguyen et al, 2021 (17)	CLEAR	Retrospective	Mechanical thrombectomy	1604	70 [58.5-80]	52.9%	16 [12-20]	8 [7-9] (n=1547)	17.0%	22.0%	11.5 [8.3-15.0][m] (n=1584)	42.1%	21.6%	6.3% (100/1578)

Citation	Study or registry name	Study design	Treatment	No. patients	Baseline characteristics							Endpoints		
					Age, years	Female	NIHSS	ASPECTS	M2 MCA occlusion	Intravenous tPA given before procedure	Time from stroke onset to arterial puncture (unless otherwise noted), h	mRS 0-2 at 90 days	All-cause mortality at 90 days	sICH
Nogueira et al, 2018 (7)	DAWN	RCT	Stent retriever thrombectomy + medical management	107	69.4 (14.1)	60.7%	17 [13-21]	NR	1.9%	4.7%	12.2 [10.2-16.3][n] 4.8 [3.6-6.2][o]	48.6%	18.7%	5.6%
			Medical management	99	70.7 (13.2)	48.5%	17 [14-21]	NR	3.0%	13.1%	13.3 [9.4-15.8][n] 5.6 [3.6-7.8][o]	13.1%	18.2%	3.0%
			P value	---	NR	NR	NR	NR	NR	NR	NR	>0.99[p]	NSD	NSD
Nogueira et al, 2021 (22)	Trevo	Retrospective analysis of prospectively collected data	Stent retriever thrombectomy	67	65.8 (15.1)	41.8%	16 [9-20]	8 [7-9]	13.4%	23.9%	9.3 [6.8-14.5][m]	60.6% (40/66)	9.0%	1.5%
				180	66.7 (14.5)	58.9%	15 [10-19]	8 [7-9]	19.4%	26.8% (48/179)	10.2 [7.5-14][m]	54.7% (98/179)	11.1%	0.6%
Olthuis et al, 2023 (26)	MR CLEAN-LATE	RCT	Endovascular treatment[q] + medical management	255	74 [64-80]	58.0%	10 [6-17]	9 [7-10]	31.4%	4.7%	11.8 [9.0-15.2]	39.2%	24.3%	6.7%
			Medical management	247	74 [64-81]	45.7%	10 [6-18]	8 [7-9]	30.8%	7.7%	11.4 [8.9-14.8]	34.0%	30.0%	1.6%
			Adjusted odds ratio (95% CI)	---	NR	NR	NR	NR	NR	NR	NR	1.54 (0.98-2.43)	0.72 (0.44-1.18)	4.59 (1.49-14.10)
Santos et al, 2019 (13)	-----	Prospective observational	Aspiration or stent retriever thrombectomy	63	73; range, 59-80	54.0%	16 [12-18]	8 [7-9]	20.6%	34.9%	8.6 [7.1-13.6][m]	65.1%	7.9%	3.2%

3D, 3D Revascularization Device; ADAPT, A Direct Aspiration First Pass Technique; AIS, acute ischemic stroke; ASPECTS, Alberta Stroke Program Early CT Score; LVO, large vessel occlusion; MCA, middle cerebral artery; mRS, modified Rankin Scale score; NIHSS, National Institutes of Health Stroke Scale score; NR, not reported; NSD, no significant difference; RCT, randomized clinical trial; sICH, Symptomatic intracranial hemorrhage; tPA, tissue plasminogen activator.

[a]Time from stroke onset to randomization.
[b]For patients with a witnessed stroke onset, the median time to randomization was 9.5 hours.
[c]Time from symptom onset to presentation or, if that time was not available, time from last well known to presentation.
[d]M1 or all M2 MCA segments, 20.9% (21/31); single M2 MCA segment, 3.2% (1/31).
[e]M1 or all M2 MCA segments, 73.1% (19/26); single M2 MCA segment, 0.0% (0/26).
[f]Patients with a time from last seen healthy to randomization of 5.5 and 12 hours were presumed to have a time from last seen healthy to arterial puncture of greater than 6 hours, as the 75th percentile time from randomization to arterial puncture was 28 minutes.
[g]ASPECTS not measured in 49 patients.
[h]*P* value for adjusted odds ratio.
[i]Patients selected according to DEFUSE-3 criteria.
[j]56% of patients in the entire GSR-ET registry of a similar size (27).
[k]Numerator and denominator not reported.
[l]Patients selected according to DAWN criteria.
[m]Time from last seen well to puncture.
[n]Time from patient last known well to randomization.
[o]Time from first observation of symptoms to randomization.
[p]Posterior probability of superiority.
[q]Any CE-marked endovascular treatment device.
